# Understanding the sexual recruitment of one of the oldest and largest organisms on Earth, the seagrass *Posidonia oceanica*

**DOI:** 10.1371/journal.pone.0207345

**Published:** 2018-11-16

**Authors:** Laura Guerrero-Meseguer, Carlos Sanz-Lázaro, Arnaldo Marín

**Affiliations:** 1 Departamento de Ecología e Hidrología. Facultad de Biología, Universidad de Murcia, Campus de Espinardo, Murcia, Spain; 2 Departamento de Ecología, Pabellón 13, Universidad de Alicante, Alicante, Spain; Universita degli Studi di Genova, ITALY

## Abstract

The seagrass *Posidonia oceanica* is considered one of the oldest and largest living organisms on Earth. Notwithstanding, given the difficulty of monitoring its fruits and seeds in the field, the development of *P*. *oceanica* during its sexual recruitment is not completely understood. We studied the stages of development of *P*. *oceanica* seeds from their dispersion in the fruit interior to their settlement in sediment through histological, ultrastructural and mesocosm experiments. *P*. *oceanica* sexual recruitment can be divided into three main stages that focus on maximising photosynthesis and anchoring the seedlings to the sediment. In the first stage (fruit dispersion), seeds perform photosynthesis while being transported inside the fruit along the sea surface. In the second stage (seed adhesion), seeds develop adhesive microscopic hairs that cover the primary and secondary roots and favour seed adhesion to the substrate. In the last stage (seedling anchorage), roots attach the seedlings to the substrate by orienting them towards the direction of light to maximise photosynthesis. The adaptations observed in *P*. *oceanica* are similar to those in other seagrasses with non-dormant seeds and fruits with membranous pericarps, such as *Thalassia* sp. and *Enhalus* sp. These common strategies suggest a convergent evolution in such seagrasses in terms of sexual recruitment. Understanding the sexual recruitment of habitat-forming species such as seagrasses is necessary to adequately manage the ecosystems that they inhabit.

## Introduction

Seagrasses are formed by a polyphyletic group of monocotyledons (order Alismatales), which recolonised marine environments 80 million years ago [1]. Seagrasses are habitat-forming species because they are a source of food and shelter for a wide variety of fish and invertebrates, and they perform relevant ecosystems services [2,3]. Despite their importance, seagrass populations are currently threatened by a variety of anthropogenic stressors [4,5]. The ability of seagrasses to cope with environmental perturbations depends, to some extent, on genetic variability, which is obtained through sexual recruitment [6–8]. By forming new individuals, seagrasses increase their genetic diversity and thus their ability to colonise new areas and to adapt to environmental changes [9–13].

Seagrasses have contrasting colonisation strategies [14]. Some seagrasses form seed banks of small seeds with hard pericarps that can remain in the dormancy stage for several months. These seagrasses are generally short-lived and can recover quickly from disturbances by not germinating far away from parent meadows (e.g., *Halophyla sp*., *Halodule sp*., *Cymodocea sp*., *Zostera sp*. and *Heterozostera sp*. [14,15]). In contrast, other seagrasses form dispersal propagules. This strategy is typical of long-lived seagrasses that can form buoyant fruits with inner large non-dormant seeds, such as the genera *Posidonia sp*., *Enhalus sp*. and *Thalassia sp*. [14,16]. Accordingly, the seeds of long-lived seagrasses have a large dispersal capacity compared to the seeds of the short-lived type [17,18], which permits the evolution of species beyond unfavourable light conditions by the seedling development of parent meadows.

The seagrass *Posidonia oceanica* (L.) Delile is considered one of the oldest and largest species on Earth. An individual can form meadows measuring nearly 15 km wide and can be as much as 100,000 years old [19]. *P*. *oceanica* meadows play important roles in the maintenance of the geomorphology of Mediterranean coasts, which, among others, makes this seagrass a priority habitat of conservation [20]. Currently, the flowering and recruitment of *P*. *oceanica* seems to be more frequent than that expected in the past [21–25]. Furthermore, this seagrass has singular adaptations to increase its survival during recruitment. The large amounts of nutrient reserves contained in the seeds of this seagrass support shoot and root growth, even up to the first year of seedling development [26]. In the first months of germination, when leaf development is scarce, *P*. *oceanica* seeds perform photosynthetic activity, which increases their photosynthetic rates and thus maximises seedling establishment success [27,28]. Seedlings also show high morphology plasticity during their root system development [29,30] by forming adhesive root hairs to help anchor themselves to rocky sediments [21,31,32]. However, many factors about *P*. *oceanica* sexual recruitment remain unknown, such as when photosynthesis in seeds is active or how seeds can remain anchored to and persist on substrate until their root systems have completely developed. Increasing our knowledge about *P*. *oceanica* adaptations during sexual recruitment is essential to design environmental policies that conserve threatened habitat-forming seagrasses with similar characteristics.

The objective of this study was to increase our understanding of the morphological and physiological adaptations involved in the dispersion and settlement of seagrasses that form non-dormant seeds and buoyant fruits. Using *P*. *oceanica* as a model of this type of seagrass, we performed a histological analysis and mesocosm experiments to evaluate the importance of light and substrata type in the first weeks of sexual recruitment.

## Materials and methods

### Fruit collection and seed germination

*Posidonia oceanica* sexual recruitment was studied by defining the dispersion and settlement stages and analysing the fruit pericarp, newly released and 1-week-old seeds (S1 Fig). Seed development in the dispersion stage was evaluated by performing histological and light-transmission analyses in the fruit pericarps and by testing photosynthetic activity in the newly released seeds. The settlement stage was evaluated by performing ultrastructural analyses in the primary system of seed adherence, and two mesocosm experiments in which the influences of light and type of substrata on the development of the 1-week-old seeds were tested. Finally, the primary root system morphology and the process of anchorage in the seedlings were analysed after two months of development.

**Fig 1:**
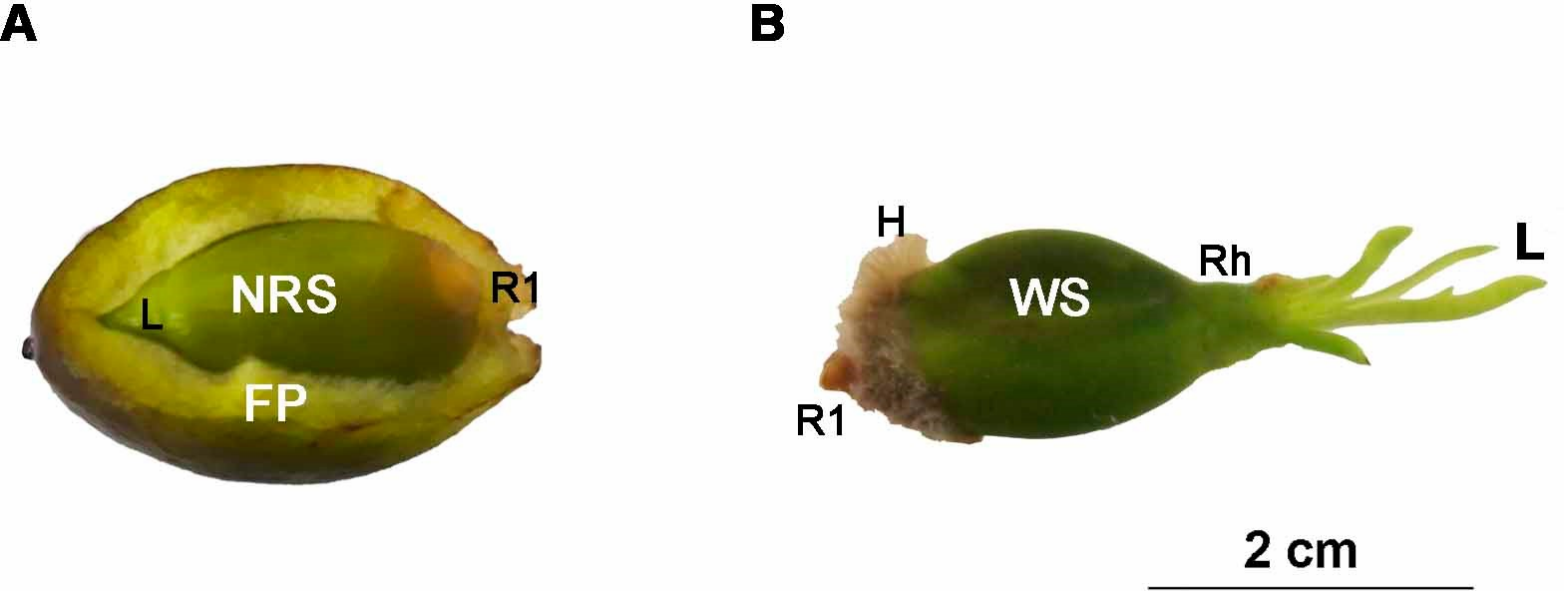
Newly released and 1-week-old seeds of *P*. *oceanica*. (A) Newly released seeds inside a fruit and (B) 1-week-old seeds of *Posidonia oceanica*. FP, fruit pericarp; NRS, newly released seeds; WS, 1-week-old seeds; H, adhesive hairs; S, seed; R1, primary root; Rh, rhizome; L, leaves.

*Posidonia oceanica* fruits were collected on beaches in the Murcia Region (Spain) in May 2016 under the authorisation of the Autonomous Spanish Community of the Murcia Region. In this area, *P*. *oceanica* meadows are mainly found on the sandy beaches between 1 and 25 m depth [33]. Exceptionally, flowering and seed production have occurred in this area during the last four years. To ensure that the fruits used in the experiments were floating for a similar period of time, only immature and healthy fruits (with closed and dark green pericarps) were collected. The fruits were ovoid (2.54±0.21 cm long and 1.60±0.05 cm wide), and their pericarps resembled a membranous coating over the seeds (Fig 1A). The fruits were immediately transported to the laboratory to avoid pericarps degradation and were placed inside aquaria filled with artificial seawater and sufficient aeration to continuously maintain an oxygen concentration above 5.5 mg O_2_/l^-1^. Then, fruits were separated from the seeds and selected for the experiments. To check the buoyancy of the fruit pericarps and seeds, their density was measured by calculating their wet weight and the volume they occupied in seawater.

Seeds were extracted from the fruits by longitudinally cutting the pericarp with a scalpel (newly released seed). The newly released seeds were 1.65±0.05, 0.98±0.03 and 0.57±0.02 cm in length, width and thickness, respectively, and they weighed 0.77±0.04 g (DW). The seeds were green, ovoid and, on occasion, presented a short leaf and root primordial on the apical extreme (Fig 1A). Considering that fruits with the seeds of *P*. *oceanica* can float for one to two weeks [34,35], 75 seeds were germinated for 1 week in individual glass jars filled with aerated artificial seawater to study the settlement stage (1-week-old seed, Fig 1B). The 1-week-old seeds had 3–7 leaves measuring 1.68±0.12 cm long and 0.002–0.003 cm wide. The primary root of the 1-week-old seeds ranged from 0.001 cm to 0.005 cm in length. Prior to primary root development, the base of the posterior extreme of the 1-week-old seeds developed a dense material of adhesive hairs that covered the primary root (Fig 1B).

The temperature and salinity of the seawater used in all experiments were 21°C and 36, respectively. Temperature was monitored by dataloggers (HOBO, Bourne, MA, USA). Artificial seawater was prepared with bidistilled water and marine salt (Ocean Fish, PRODAC International, Cittadella, Italy). Aeration was provided by a system of tubes and capillaries connected to an air pump. In the experiments that lasted more than 1 month, a 14:10 (light:dark) photoperiod was applied in the environmental chambers. In all the other experiments, no photoperiod was applied because the experiments lasted for less than 1 day.

### Histology analyses and light-transmission tests in the fruit pericarp

First, to analyse the cellular structures of the *P*. *oceanica* fruits, the layers that composed the fruit pericarp were observed by optical microscopy (n = 3). The fruit pericarp pieces were previously cut with a microtome (RM 2155 Leica, Leica Biosystems, Wetzlar, Germany) in 10 μm-thick sections. These sections were stained with 0.05% toluidine blue and mounted in DPX medium (Agar Scientific, Stansted, UK). The sections were observed under an optical microscope (Leica DMLB; Leica Biosistems, Wetzlar, Germany), and photographs were taken (Leica DC500, Leica Biosistems, Wetzlar, Germany).

Subsequently, to test the light that was transmitted through the pericarp to the seed, the fruit pericarp fragments were exposed to a gradient of light irradiance (from 10 to 1,000 μE· m^-2^ · s^-1^, n = 18). The light intensity capable of penetrating the pericarp was determined by placing pericarp segments (1.5 cm^2^) over the photoradiometer datalogger probe (DO 9721, Delta OHM, Padova, Italy). Then, pericarps were exposed to eight light intensities (10, 30, 50, 150, 300, 500, 750 and 1,000 μE· m^-2^ · s^-1^) by varying the distance from pericarp to the light source (LED: 20 W, 6,400 K and 1,600 lumens daylight; Electro DH, Barcelona, Spain). To ensure a similar area of light availability in all the measurements, the probe was covered by a handmade mould made of dark plasticine with a 1 cm-diameter gap left in the middle (S2 Fig). The employed light intensities were based on the average solar light radiation recorded in the month of fruit dispersion (May) on the Murcia Region coast (1,035.6±23.79 μE· m^-2^ · s^-1^; data download from the Agricultural Information System of the Murcia Institute for Agricultural and Food Research and Development, IMIDA; http://siam.imida.es/).

### Photosynthetic activity tests

Photosynthetic activity was tested in the fruits and seeds with their corresponding light intensities. Light-adapted yield and rapid light curves (RLCs) were also measured in the fruit pericarps and in both seed development stages by PAM fluorometry (MINI-PAM, Waltz, Effeltrich, Germany).

Net primary production (NPP) and respiration rates were tested by measuring the oxygen concentration (optical electrode; Portable Meter Hach HQ30d, HACH, Loveland, Colorado, USA) in the dark and light incubations of the fruit pericarps, newly released and 1-week-old seeds separately (n = 5). Then, gross primary production rates (GPP) were calculated by subtracting the respiration from the NPP rates. Incubations were carried out in airtight glass jars filled with artificial seawater at the average light intensities obtained during the *P*. *oceanica* fruit dispersion period (~1,000 μE· m^-2^ · s^-1^) and at the average light radiation that seeds reached after passing through the pericarp (~10 μE· m^-2^ · s^-1^).

RLCs were performed in the fruit pericarps and seeds using a range of light intensities from 10 to 1,000 μE· m^-2^ · s^-1^ (n = 5). Each light intensity was applied for 10 s, which was followed by a saturating pulse of 0.2 s. The *r*ETR values against light irradiances were fit to the exponential model proposed by Platt et al. 1980 [36]. The derived parameters of RLCs, including photosynthetic efficiency (*α*), photoinhibition parameter (*β*), maximum electron transport rate (*r*ETR_max_) and saturation irradiance (E_k_), were calculated following the equation of Ralph & Gademann 2005 [37]. Additionally, light-adapted yields were tested in the seeds (n = 5) to verify whether photosynthesis efficiency depended on the prior adaptation to light [38].

### Influence of light on the seed settlement stage

To evaluate the influence of the direction of light on the settlement stage, the 1-week-old seeds (n = 8) were incubated according to three different directions of light with respect to the longitudinal seed axis (top, right or left) for 2 months. To determine if the grain size of the substrata and the light direction had interactive effects on seedling responses, two types of substrata (sand and pebble) were used in the experiment.

The three directions of light were applied by using three environmental chambers: an environmental chamber with the light bulbs placed on the roof and two environmental chambers with the light bulbs positioned on walls. The seeds were placed individually in autoclaved glass jars filled with artificial seawater and substrate (2 cm). The seeds were oriented with the apical extreme so that leaves emerged and faced the back of the environmental chamber. The sand used in the experiments was collected at the same beach as the fruits. The pebble was a sterilised substrate for aquariums (Akvastabil, Haderslev, Denmark). The grain size of the sand was 0.03% gravel, 1.46% very coarse sand, 85.1% coarse sand, 13.4% medium sand and 0.01% fine sand. The grain size of the pebble substrate was 80.8% pebble and 19.2% gravel [39]. The light intensity on the seeds was 100 μE· m^-2^ · s^-1^.

After 2 months, the angle of orientation of the seeds was measured in regard to the light direction: above (π/2 radians), right (2π radians) and left (π radians). Then, the final angle of seed rotation obtained in each treatment was subtracted from the initial angle of seed orientation (π/2 radians).

### Ultrastructure of the adhesive hairs

Prior to primary root development, the morphology of the adhesive hairs in the 1-week-old seeds (n = 3) was analysed by transmission and scanning electron microscopy.

For transmission electron microscopy (JSM 6100, Jeol, MA, USA), pieces of the basal surface of the 1-week-old seeds and primary roots were fixed in 2.5% Milloning´s phosphate-buffered glutaraldehyde (pH 7.2–8.2) for 1 h. These pieces were washed in 2.5% NaHCO_3_ (60 min at 25°C) and post-fixed in a solution of 2% OsO_4_ and 1.25% NaHCO_3_ for 1 h. Subsequently, the pieces were dehydrated in an ethanol series and embedded in an epoxy resin solution (Epon). Then, ultra-thin transverse sections were cut with glass and diamond knives. Sections were stained in a solution of uranyl acetate and lead citrate before being observed under a microscope.

For the scanning electron microscope observations (JEOL-6100 Scanning Microscope; Oxford Instrument, Abingdon, UK), the pieces of the basal surface of the seeds and the pieces of their primary roots were previously dehydrated in 96% absolute ethanol and then point-dried and sputter-coated with gold.

### Influence of substrata type on the seed settlement stage

To evaluate the influence of substrata type on the success of anchorage and the ultrastructure of the root system, 1-week-old seeds were individually placed into glass jars filled with artificial seawater and substrata (sand, pebble, sand+pebble and fibreglass; n = 8). The sand and pebble treatments comprised the substrata used in the previous experiment. The sand+pebble treatment involved mixing 50% of the sand and 50% of the pebble from the previous treatment. The fibreglass treatment was used to evaluate the effects of a fibrous substrate on the root system morphology, such as a canopy of algae or a surface composed of seagrass beach-casts with no organic matter decomposition. The light intensity was 50 μE· m^-2^ · s^-1^ during daylight hours.

After the first month of the experiment, the success of the root system anchorage and the presence of adhesive hairs were estimated by ranking them into percentages depending on the number of roots anchored and hair density, respectively (S1 Table). Finally, after 2 months, the root system of three samples per treatment was observed by scanning electron microscopy.

### Data analysis

The gradient of light transmitted through the fruit pericarp was fitted to a regression model, which was chosen with the correlated Akaike information criterion test (AICc).

One-way ANOVAs were used to test the differences between the treatments in photophysiological parameters (*α*, *β*, *r*ETR_max_, E_*K*_ and light-adapted yield) and photosynthetic activity (NPP, GPP and respiration). Prior to ANOVA, data were tested for normality and homoscedasticity of variances by the Shapiro and Bartlett tests, respectively. Transformations were applied if data did not meet the assumptions. Statistical tests were conducted with a significance level of *α =* 0.05. In those cases in which data did not meet the assumptions after being transformed, the significance level was lowered to *α =* 0.01 [40]. Tukey’s test was used to examine the pairwise differences among levels when the main effects showed significant differences. The influence of the substrata type on the seed anchorage success and the density of adhesive root hairs were tested by applying Kruskal-Wallis and Kramer (Nemenyi) tests.

To test the influence of the direction of light during seedling settlement, a Watson–William’s test was used to determine whether the mean of the angles obtained in the seeds cultured under lateral light (right and left) differed from the mean of the angles obtained in the seeds grown with overhead light in both substrata types used in the experiment (sand and pebble). Rayleigh’s tests for circular uniformity were previously tested to determine that data were unimodal and not diametrically bidirectional.

Statistical analyses were performed with the R statistical software (v. 3.2.5) using the packages “AICcmodavg”, “GAD”, "PMCMR" and “CircStats” [41]. The data results are reported throughout the manuscript as the mean ± standard error (SE).

## Results

### External morphology and the fruit pericarp ultrastructure

**Fig 2:**
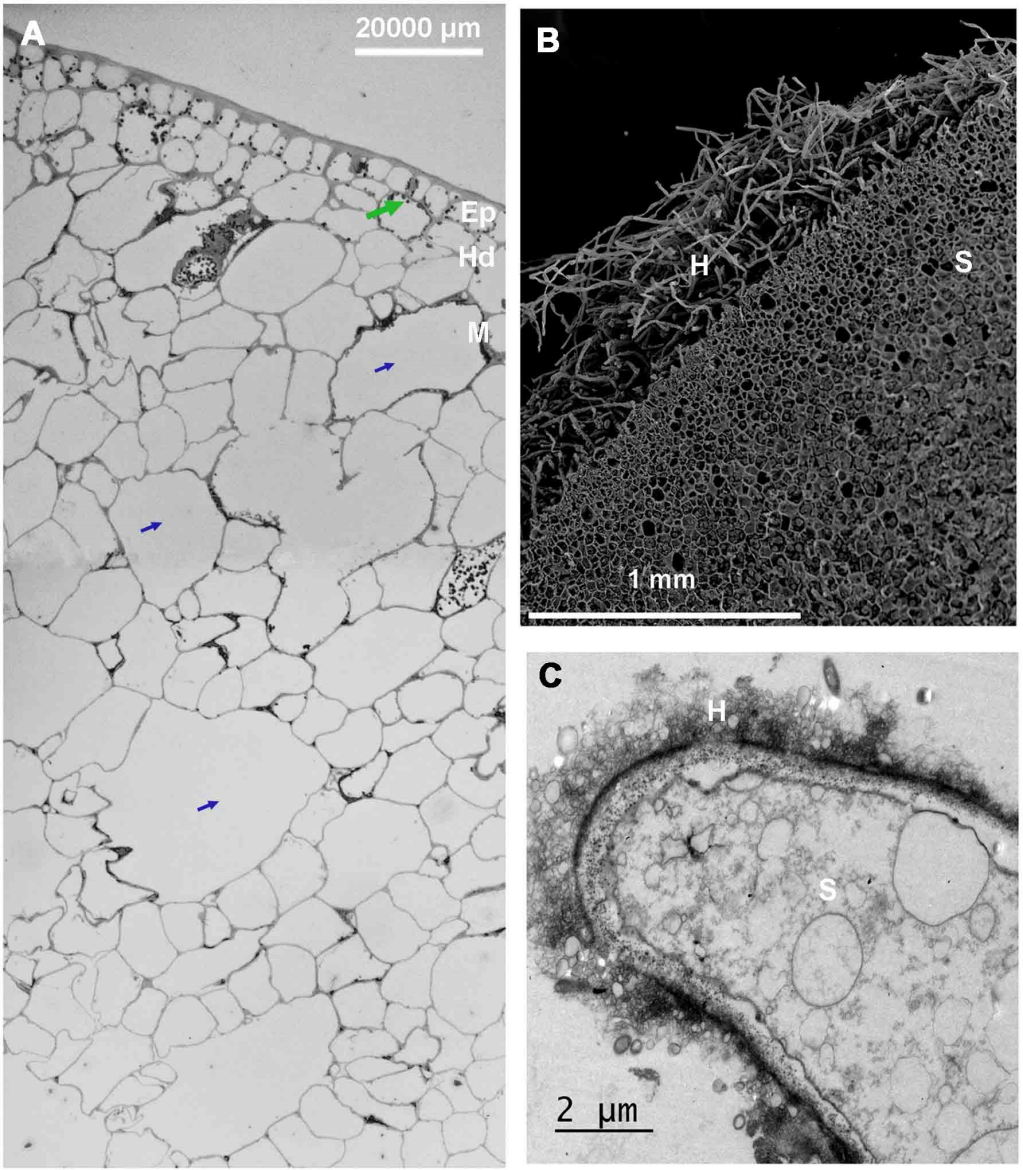
Details of the histology, ultrastructure and morphology of the fruit pericarp and 1-week-old seeds of *Posidonia oceanica*. Images show (A) the histological fruit pericarp sections and (B and C) the ultrastructure of the adhesive basal hairs of a 1-week-old seed. Green arrows indicate chloroplasts, while blue arrows denote air lacunae. Ed, epidermis; Hd, hypodermis; M, mesophyll; H, adhesive hairs; S, seed.

The histological analyses showed that the *P*. *oceanica* fruit pericarps displayed the typical fruit covering structure formed by an initial layer of epidermis and a subsequent layer of mesophyll (Fig 2A). The epidermis consisted of a single layer of thick-walled, rounded and relatively large epidermal cells (4,023.3±219.3 μm) covered by a cuticle (523.3±83.5 μm), with the cytoplasm showing numerous chloroplasts. The mesophyll consisted of two layers of different cell types, the hypodermis and the spongy mesophyll. The hypodermis (11,502.6 ± 1,990.5 μm) consisted of a compact coat of hexagonal-shaped cells with electro-dense material, and chloroplasts were distributed in the cytoplasm periphery (Fig 2A). The spongy mesophyll (149,987.7±3,400.8 μm) comprised large cells containing central vacuoles or air lacunae that occupied the main cellular volume (Fig 2A). The volume of the central vacuoles or air lacunae increased toward the internal mesophyll (from 1 to 3.5 mm in diameter). The fruit pericarp and the newly released seed densities were 716.9±64.1 and 1,073.0±18.9 kg^-1^ · m^-3^, showing positive and negative buoyancies in seawater, respectively (density of the seawater ~ 1,025 kg^-1^ · m^-3^).

### Light transmission in the fruit pericarps

**Fig 3:**
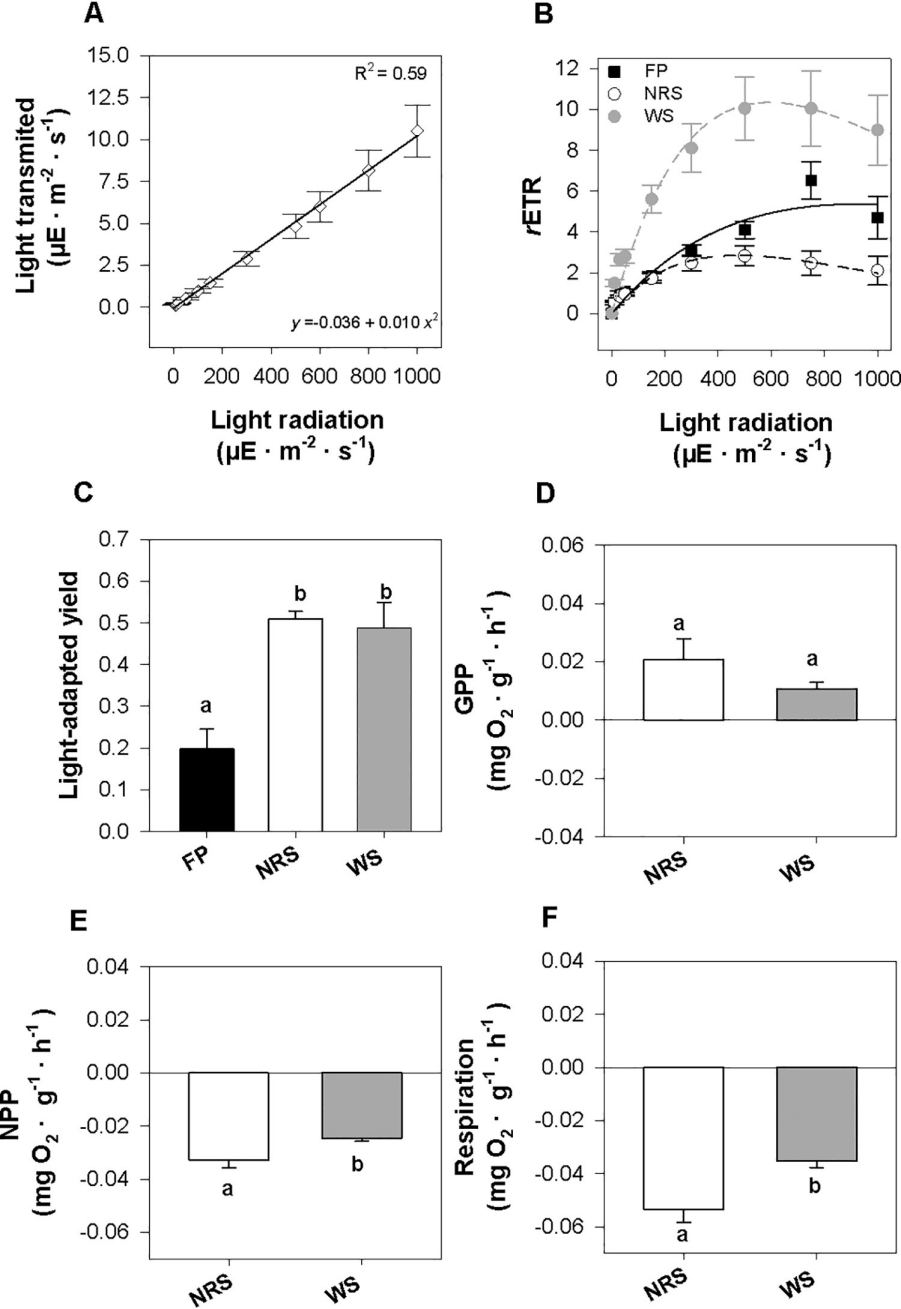
Influence of light on the sexual recruitment of *P*. *oceanica*. The graphs show the results obtained in the experiments performed to test the influence of light on the sexual recruitment of *Posidonia oceanica* (mean±SE; n = 5): (A) light transmitted by the fruit pericarps in a gradient of light irradiance; (B) RLCs; (C) light-adapted yields of the fruit pericarp and seeds; (D) GPP; (E) NPP; and (F) respiration obtained in newly released (NRS) and 1-week-old seeds (WS) at a light intensity of 10 μE · m^-2^ · s^-1^. Lines indicate significant regressions (*p*< 0.01) in the fruit pericarps (black lines; R^2^ = 0.98), newly released seeds (dotted black lines; R^2^ = 0.96) and 1-week-old seeds (dotted grey lines; R^2^ = 0.97). Letters indicate significant pairwise differences between the treatments.

The light transmission through the pericarps followed a significant linear trend according to the light intensity exposure (Fig 3A). The light transmission in the pericarps ranged between 0.14±0.01 and 10.50±1.56 μE· m^-2^ · s^-1^ for light exposures of 10 to 1,000 μE· m^-2^ · s^-1^, respectively (Fig 3A).

### Photosynthetic activity in the fruits and seeds during dispersion

The RLCs showed significant differences in the parameters of *α* and *r*ETR_max_ among the fruit pericarps, the newly released seeds and the 1-week-old seeds (Table 1 and Fig 3B). The highest *α* values were found for the 1-week-old seeds (0.048 ± 0.005), while the fruit pericarps and the newly released seeds gave similar values (0.016±0.001 and 0.017±0.002 in the fruit pericarps and the newly released seeds, respectively; Table 1 and Fig 3B). The newly released seeds and the 1-week-old seeds had significantly higher *r*ETR_max_ values (6.207±1.484 and 10.41±1.64, respectively) than the seeds obtained in the fruit pericarps (2.905±0.496; Table 1 and Fig 3B). Notwithstanding, the *β* and E_*K*_ values between treatments were similar (Table 1).

**Table 1:** Summary of the results obtained by one-way ANOVA and Tukey's HSD test of the photophysiology parameters (α, β, *r*ETRmax, E_K_ and light-adapted yield) of the fruit pericarps (FP), newly released (NRS) and 1-week-old seeds (WS) of *Posidonia oceanica* (n = 5). The numbers in bold indicate significant effects (*p* <0.01). An asterisk over the response variable indicates that the data did not meet the assumptions, and a significance level of α = 0.01 was applied.

	One-way ANOVA	Pairwise comparisons		
Response variable	*p*	FP vs. NRS	FP vs. WS	NRS vs. WS
		*p*	*p*	*p*
*α*	**< 0.01**	0.998	**< 0.01**	**< 0.01**
*β*	0.078	0.065	0.425	0.460
*r*ETR_max_	**< 0.01**	0.216	0.099	**< 0.01**
E_K_	0.077	0.088	0.155	0.938
Light-adapted yield	**< 0.01**	**< 0.01**	**< 0.01**	0.943

The light-adapted yield in the fruit pericarps was significantly lower than that in the newly released and 1-week-old seeds (Fig 3C). At a light intensity of 1,000 μE ·m^-2^·s^-1^, the fruit pericarps obtained negative values for NPP (-0.272±0.014 mg O_2_ · g^-1^· h^-1^) and respiration rates (-0.234±0.007 mg O_2_ · g^-1^· h^-1^), which gave a negative GPP rate (-0.039 ± 0.017 mg O_2_· g^-1^· h^-1^). At 10 μE ·m^-2^·s^-1^ of light intensity, the seeds had similar positive GPP values (*p* = 0.437; Fig 3D). In contrast, the newly released seeds showed significantly lower values for respiration and NPP than the 1-week-old seeds (*p*< 0.05; Fig 3E and 3F).

### Influence of the direction of light on the seed orientation

The 1-week-old seeds showed a positive phototropism to the direction of light (S3 Fig). The angles of orientation of the seeds grown with light on the walls significantly differed from the angles of the seeds that developed with light that came from overhead (*p*< 0.01). When light came from overhead, seeds turned an average of 0.061±0.019 radians in relation to the initial position. Seeds rotated an average of 0.878±0.046 radians when the light came from the right and 2.242±0.041 radians when the light came from the left (S3 Fig). However, the phototropism of seeds was not influenced by the sediment type in which the seedlings were grown (sand and pebble; S3 Fig).

### Primary system of seed adherence

**Fig 4:**
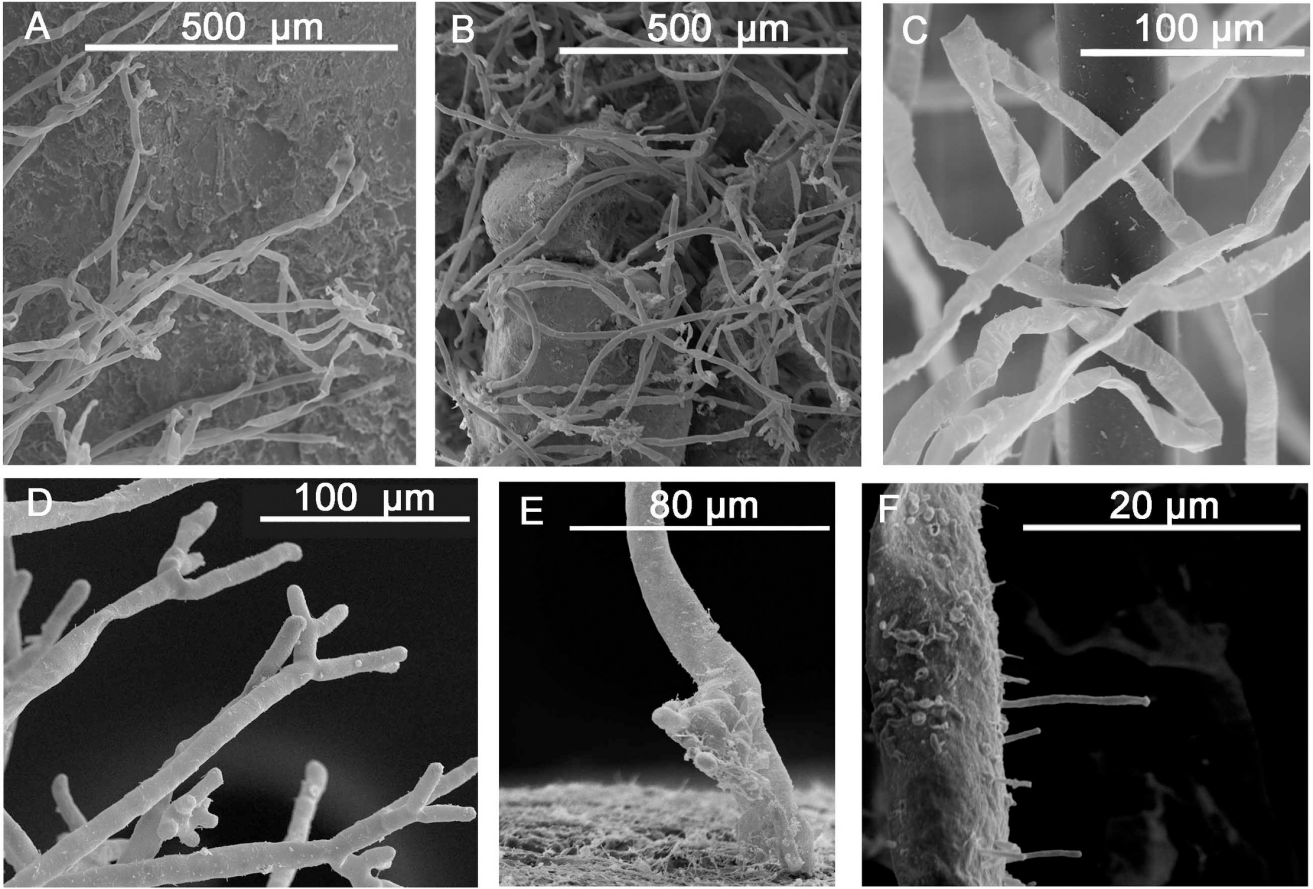
Root hair ultrastructure of *P*. *oceanica*. Images obtained by scanning electron microscopy of the root hairs of the *Posidonia oceanica* seedlings grown in different types of sediment treatments: (A) root hairs adhered by their basal extreme in the pebble treatment, (B) root hairs surrounding grains of sand in the sand treatment, (C) root hairs that interwove a fibre in the fibreglass treatment, (D) the branched edges of root hairs, (E) the basal extreme of a root hair anchored to the pebble treatment and (F) the microvillus of the lateral surface of root hairs.

After approximately 1 week of development, a matrix of adhesive hairs appeared on the basal surface of the seeds (Fig 2B and 2C). In subsequent weeks, the adhesive hairs concentrated on the most posterior seed part, covering the primary root (Fig 1B). After 1 month of development, all the seeds, primary roots and secondary roots had adhesive hairs over their entire surfaces. The adhesive hairs disappeared from the surface of seeds after 2 months of development but persisted in the primary and secondary roots for the rest of the experimental period (Fig 4).

The adhesive hairs of the seeds had the same structure as the adhesive hairs of the primary and secondary roots of the seedlings (Fig 2B and Fig 4). On both primary and secondary roots, the adhesive hairs came from hypodermis cells, were tubular-shaped, and covered a seed length from 5 to 10 μm and a seed width from 0.5 to 1 μm (Fig 2B and 2C). The lengths of the root hairs were not measured, as they were too entangled. The root hairs were highly branched (Fig 4D) and produced microtrichomes over their lateral sides (Fig 4F).

### Influence of substrata type on the root system morphology

The root system anchoring success was not significantly different between the substrata types (Table 1). Nevertheless, the lowest anchorage success was seen in the sand treatment (45.0±14.6%), while the highest successes were seen in the fibreglass, pebble and sand+pebble treatments (100±0.0%, 95.0±5.0% and 90.0±6.1%, respectively).

Substrate type did not influence the production of adhesive hairs in either seeds or roots (Table 2). The presence of hairs was clearly noted, and hairs formed a layer over the seeds and primary roots, which was slightly greater in the roots (56.0±6.1%) than that in the seeds (48.0±11.4%). Hair length was not measured in this experiment because the hairs were completely incrusted or wrapped in sediment (see Fig 4A, 4B and 4C). The root hairs of the seedlings in the sand or fibreglass treatments were enmeshed with their respective substrata (Fig 4B and 4C). In contrast, in the seeds grown in pebbles, the root hair edges were anchored to the surface of the pebbles to form an amorphous adhesive matrix (Fig 4A and 4E). The seedling root system formed a tripod-like structure made up of elongated secondary roots, even in the seedlings that were not completely anchored to the substrata (S4 Fig)

**Table 2:** Summary of the statistical results obtained by the Kruskal-Wallis and Kramer (Nemenyi) tests on the effect of the substrata type on the root system morphology of *Posidonia oceanica* (n = 5). The numbers in bold indicate significant effects (*p* < 0.01). S: sand treatment, S+P: sand+pebble treatment, P: pebble treatment and F: fibreglass treatment.

		FPairwise comparisons					
			S vs. S+P	S vs. P	S vs. F	S+P vs. P	S+P vs. F	P vs.
	*p*	*p*	*p*	*p*	*p*	*p*	*p*
Anchorage success	0.02	0.38	0.18	0.07	0.97	0.83	0.97
Seed adhesive hairs	0.48	0.79	0.99	0.99	0.61	0.59	1
Root hairs	0.86	0.94	1	1	0.88	0.94	1

## Discussion

**Fig 5:**
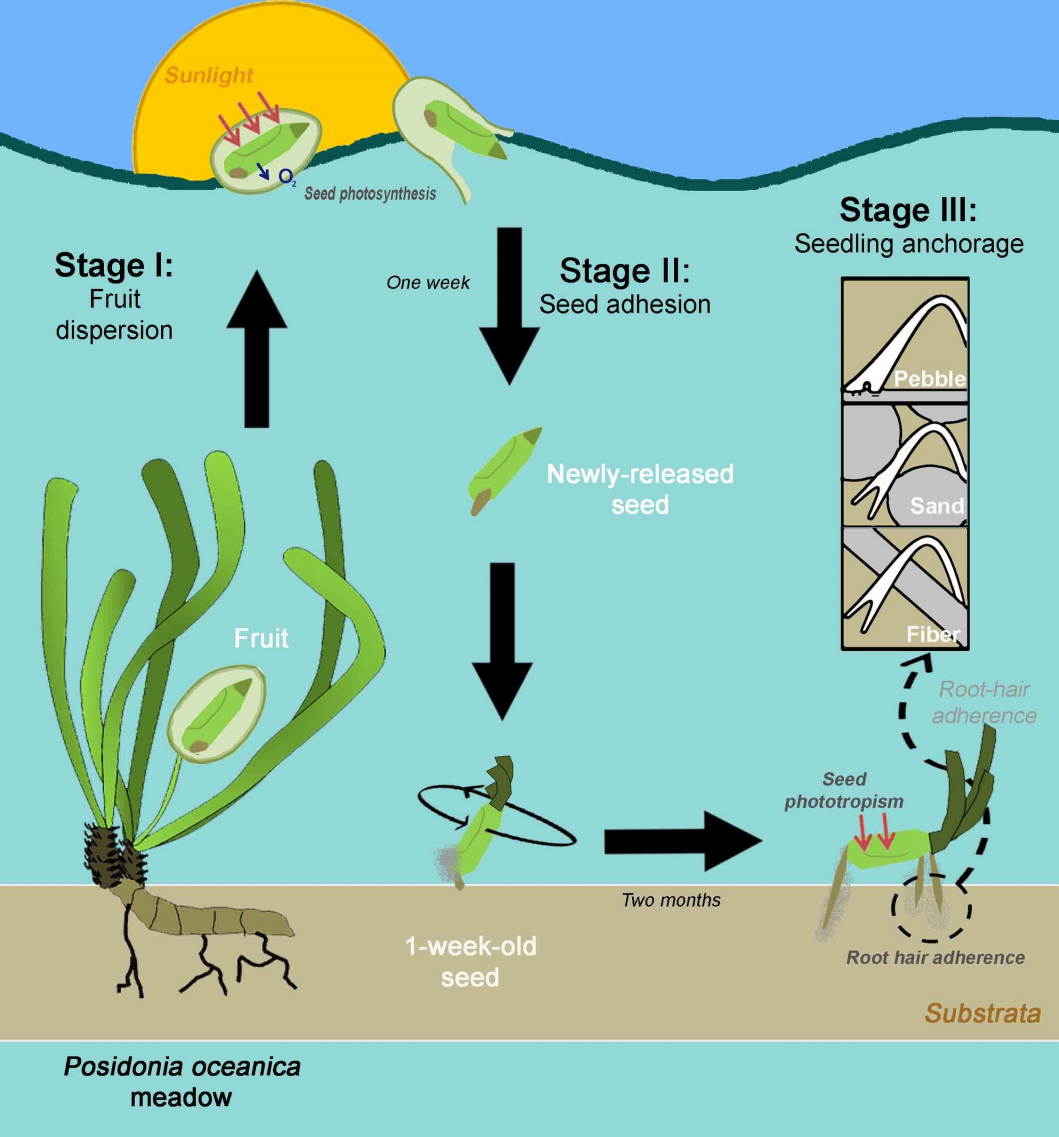
Schematic representation of the three sexual recruitment stages of *P*. *oceanica*, including dispersion and settlement.

Our results shed light on the development of *P*. oceanica seeds during sexual recruitment. Based on this and previous knowledge, we divided the process into three stages (Fig 5): *(I) fruit dispersion*, where the seeds displayed relevant photosynthetic activity inside the fruits; *(II) seed adhesion*, where the seeds developed adhesive hairs on their basal surfaces and primary roots; and *(III) seedling anchorage*, where the seeds produced a tripod-like form with their primary and secondary roots and oriented themselves to face light. These three developmental stages of sexual recruitment focus on two common colonisation strategies: maximisation of the photosynthesis of the seeds and enhanced seed anchorage to the substrate.

### Stage I: Fruit dispersion

*Posidonia oceanica* seeds are formed inside large ovoid fruits consisting of a spongy pericarp. The fruit pericarp confers buoyancy while favouring light transmission to seeds. The seeds are also capable of performing relevant photosynthetic activity while being transported to the fruit interior (Fig 5).

The chloroplasts in *P*. *oceanica* fruits are mainly distributed on the outermost pericarp layers. These chloroplasts obtained high electron transport rates at the expected light intensities on the Mediterranean Sea surface during the fruit dispersion period of *P*. *oceanica*. This finding suggests that the fruits can use light that reaches the surface of the sea to produce oxygen. In other plants, whose seeds are also dispersed by floating in aquatic environments, the oxygen produced by pericarps in the dispersion stage is essential to maintain healthy fruit tissue by maximising buoyancy in water and thus favouring dispersion [42]. The respiration rates of the *P*. *oceanica* fruits were very high, causing negative values of GPP. This fact was also reflected by the low light-adapted yield values obtained in the fruit pericarps and indicates poor photosynthesis efficiency in the photosystems. In contrast, the seeds were able to produce a positive GPP while showing high light-adapted yield values at low light intensities. In addition, the air lacunae of the pericarp (located near the seed position) increased in size in the innermost mesophyll areas. Therefore, the seeds’ efficient photosynthesis activity, despite the low light intensity, indicated that the seeds were the main contributors to pericarp oxygenation.

In this stage, the newly released seeds displayed a similar photosynthetic efficiency to that in fruits for 1 week. However, at the same light intensities, the younger seeds had lower electron transport rates than the older seeds. The respiration rates were higher in the newly released seeds than those in the 1-week-old seeds, probably due to the low light intensity inside the fruit. High respiration rates resulted in negative NPP rates, despite the seeds producing positive GPP rates in both seed development stages. Therefore, the benefits of a positive GPP in seeds can be 2-fold, i.e., slowing down pericarp degradation and favouring seed dispersion, but a positive GPP can also enhance initial seed development inside the fruit. Part of the energy that seeds obtain through photosynthesis is invested in root and leaf development in at least the first month of germination [28]. Thus, the ability of *P*. *oceanica* seeds to use low light intensities for photosynthesis could be a strategy to enhance seed development during dispersion. This strategy could explain the advanced state of germination of the seeds when released from the fruit.

### Stage II: Seed adhesion

When fruits open, seeds are released and fall to the seabed, aided by the geotropism that promotes early primary root formation. The seed forms adhesive hairs on its basal surface and primary roots, which improve adherence to the substrate. In addition, seeds increase their range of light tolerance for photosynthesis by enhancing their photosynthetic activity (Fig 5).

The high electron transport rates obtained in the RLCs of the 1-week-old seeds compared to those in the newly released seeds indicated a clear photoacclimation in the *P*. *oceanica* seeds over time [43]. Inside fruits, light intensity is low, and the newly released seeds do not need to adapt to high light intensities. In contrast, the 1-week-old seeds need to adapt to new light intensities once released. Adult *P*. *oceanica* meadows also show marked adaptations to light according to their depth [44]. Similar to adult plants, *P*. *oceanica* seeds adapt their light tolerance range to optimise photosynthesis performance during different seed development stages by enhancing their germination during settlement.

Regarding settlement, *P*. *oceanica* seeds developed adhesive hairs on their basal surfaces. The formation of adhesive hairs also occurs in other seagrasses of genera *Thalassia* and *Enhalus* but seems scarce in species of the genus *Posidonia* [45,46]. The production of adhesive hairs helps seeds and primary roots come into contact with sediment and increases the possibilities of adhesion to the substrate [47–49]. Adhesive hair formation also occurs in seagrasses that form dormant seeds and helps these seeds bury under sediment and form seed banks [50–52]. In freshwater macrophytes, such as wetland plants, adhesive basal hairs also perform important functions during seed development and water uptake before primary roots completely develop [53–55]. Hence, the formation of the adhesive hairs in seagrasses does not seem directly related to the seed dormancy type but appears to play a key role in the initial seed adherence in aquatic environments prior to full primary root development.

The histological structure of the initially formed adhesive hairs in the *P*. *oceanica* seeds is similar to that observed on the primary and secondary roots of the seedlings after 1 month of development. In both cases, the adhesive hairs were long, tubular and immersed in a gelatinous matrix. The adhesive hairs of both seeds and roots were formed from the differentiation of hypodermic cells, which also occurs in the genus *Thalassia* [48]. Therefore, the formation of the adhesive hairs on the basal surface of *P*. *oceanica* seeds and roots could be an adaptation to overcome seedling anchorage difficulties in the first weeks of development. By forming adhesive hairs on the basal surface, seeds establish contact between their primary roots and the sediment and thus maximise the anchorage capacity of the seedlings to the seabed and facilitate their geotropism.

### Stage III: Seedling anchorage

During the first month of development, seeds develop secondary roots on their apical extremes, which are later covered by adhesive root hairs. As secondary roots elongate with the primary root, they form a tripod-like structure with a two-fold function of favouring the anchorage success of seedlings to substrate and orientating seeds towards the light source (S3 and S4 Figs). This formation is linked to the presence of substrata. Increasing the light exposure of *P*. *oceanica* seeds while seedlings are anchored to a substrate could increase photosynthesis rates in the seeds by accelerating the seedling development and, thus, lead to settlement (Fig 5).

In our experiment, the secondary roots that developed in the seedlings grown in sand did not completely enter the substrate. In the other tested substrata (pebble, sand+pebble and fibreglass treatments), most seedlings anchored themselves by introducing most of their roots into the substrata. These results coincide with the good adherence capacity shown by the *P*. *oceanica* root system to rocks and substrata covered by algae [31,32,56,57]. The anchorage capacity of the seeds grown in sand could be related to the different adherence strategies observed in the root hairs. Our experiments showed that adhesive root hairs were produced in all tested substrata types. However, the root hairs seemed to use different anchorage strategies depending on the substrata type. In agreement with the results of other studies [31,58], the adhesive root hairs of the seedlings grown in pebbles adhered through their edges to the substrate and formed a kind of adhesive buttons, even when pebbles were mixed with sand. However, when the substrata were only sand or fibreglass, the root hairs did not show these adhesive buttons but embraced substrate particles instead. These different adherence mechanisms seem to be related to the presence of microtrichomes on the sides of adhesive root hairs, which could act as small hooks by sticking to grains of sand and fibres. However, the images obtained from the microtrichomes of the root hairs in this study were not sufficient to clearly understand the mechanism of adherence of the root hairs to sand and fibres. Future research in this area is needed to increase the knowledge of the functionality and mechanisms of adherence of *P*. *oceanica* root hairs in different substrata types.

Regardless of adhesion type, the *P*. *oceanica* root system seemed more stable in hard and coarse substrata than in a fine type. This coincides with *P*. *oceanica* greater sexual recruitment success on sheltered and rocky surfaces than on sand [21, 56, 57]. The high branching that showed root hairs in all sediment types could also reinforce this hypothesis. Branching increased the number of edges and the possibilities of root hairs adhering to a hard, stable substrate. In contrast, a coarse sand composition could cause grains of sand to move while roots penetrate the substrata, which would increase the possibility of uprooting seedlings during disturbance events. The seedlings of the seagrass *P*. *australis*, which usually recruits in sand, show high mortality rates during the first months of development due to grazing and bioturbation [59,60]. This phenomenon indicates that despite developing a well-adapted root system, the seagrasses of the genus *Posidonia* are very vulnerable to disturbance events when grown in substrata with a fine particle size. Thus, the high plasticity of roots to different substrata types suggests that seedling colonisation success depends more on the stability that the substrate provides to roots rather than the seedling adherence capacity.

*Posidonia oceanica* seed development strategies during sexual recruitment observed here revealed great similarity with other seagrasses that form non-dormant seeds and buoyant fruits, such as the species of genera *Thalassia* and *Enhalus*. Similar to *P*. *oceanica*, the seeds of these seagrasses are large and contain many nutrients [26,61,62], which, along with their expected photosynthetic capacity, can explain the advanced state of germination of these seeds when released from their fruits [17,47,49,63–66]. Seed photosynthetic activity has been demonstrated only in *P*. *oceanica* and *Thalassia testudinum* [27,67], but the fruits and seeds of the other species of these genera display a bright green colour on the surface, which indicates high levels of chloroplasts [48,62,68,69]. These three seagrass genera also form microscopic adhesive root hairs that enhance the anchorage of the seeds and the primary root system to substrata [31,48,58,70]. These seagrass genera also share a similar life history strategy by forming persistent extensive meadows with sporadic sexual reproduction [25]. Thus, the numerous coincidences in the adaptations of such seagrasses indicate similar sexual recruitment strategies and seem to suggest convergent evolution. Accordingly, these facts allowed us to hypothesise that this type of seagrass can have similar development stages for sexual recruitment as those described in the present study in *P*. *oceanica*. These strategies seem to be common for this type of seagrass to maximise its dispersal capacity.

The importance of light and substrate type during *P*. *oceanica* sexual recruitment demonstrated herein are important factors that need to be taken into account for the environmental management of long-lived seagrass meadows. Management decisions, such as fishing and coastal construction activities, can affect the survival of long-lived meadows, and care should be taken to not affect the sexual reproduction of seagrass meadows, especially during dispersion and settlement periods. The protection of possible recruitment areas with the necessary requirements for successful species colonisation for seagrasses is essential for the resilience of seagrass populations against present and future anthropogenic stressors. The future indicates an environmental restoration of seagrasses by way of seeds, which should take into account species substrate and environmental condition preferences during sexual recruitment. In *P*. *oceanica*, despite the growth of seeds in hard substrata providing shorter root development than the growth in sand [30], seedling anchorage success appears higher in the presence of pebbles or fibres. Such substrata types favour the adhesion and establishment of root hairs. Thus, the addition of fibrous, coarse or hard substrata to the sandy substrate generally used for *in vitro* germination, such as filamentous algae, seagrass dead matte and fragments of rocks or pebbles, could enhance the seedling anchorage success of restoration projects. Although experimental testing is needed, these environmental management recommendations could be extended to other seagrasses that form fruits with membranous pericarps and non-dormant seeds.

According to our results, *P*. *oceanica* sexual recruitment can be divided into three stages: fruit dispersion, seed adhesion and seedling anchorage. These three stages aim to maximise the recruitment success of this seagrass by promoting photosynthetic activity in the seeds and enhancing the seedling anchorage capacity to the seabed. These results also revealed the importance of environmental conditions, such as light and substrata type, for the sexual recruitment of seagrasses that form fruits with membranous pericarps and non-dormant seeds, such as *P*. *oceanica*. This knowledge should be taken into account when selecting conservation and protection areas to ensure the successful colonisation of seagrass populations. Conducting more research on the mechanisms of the adherence of *P*. *oceanica* root hairs to different substrata types and the sexual recruitment of this seagrass is absolutely necessary to improve the connectivity, genetic variability and recruitment of these important habitat-forming species.

## Supporting information

S1 Fig Schematic representation of the experimental setup used in this study. The scheme indicates the recruitment stages of the *Posidonia oceanica* seeds (seed inside fruit pericarp, newly-released and 1-week-old seeds) and the tests used in each experiment.
(TIF)

S2 Fig Photoradiometer probe covered with the handmade mould used to evaluate the light transmitted by the fruit pericarp of *Posidonia oceanica* within the gradient of light intensity. In this image, the probe, with the fruit pericarp coupled inside it, was situated 20 cm from the light source.
(TIF)

S3 Fig Test of phototropism in the *Posidonia oceanica* seeds (n = 5) grown in sand (grey circles) and pebble (white circles). Dashed lines indicate the angle of the orientation of the seeds obtained when light came from the top. Solid lines indicate the average angle of orientation of the seeds obtained in each treatment in the seedlings cultured with lateral lights (right: 180°; left: 0°). Black points indicate the angle of orientation obtained in each sample. Significant differences between the top and lateral lights (right and left) were indicated as *p <*0.01 in each treatment.
(TIF)

S4 Fig Tripod-like formation of the roots of the *Posidonia oceanica* seedlings on a sand+pebble substrate 1 month after settlement. (TIF)

S1 Table Ranges of % used in the experiment of sediment type influence on the root system morphology to determine anchorage success and the density of adhesive hairs obtained after 1 month of seedling development. (DOCX)

S1 Data Data set used in the experiments. (XLSX)
