# Supplementary figures and images for: Understanding the sexual recruitment of one of the oldest and largest organisms on Earth, the seagrass Posidonia oceanica
Source: PLoS One. 2018 Nov 16;13(11):e0207345. doi: 10.1371/journal.pone.0207345 (PMC6239318; doi:10.1371/journal.pone.0207345)

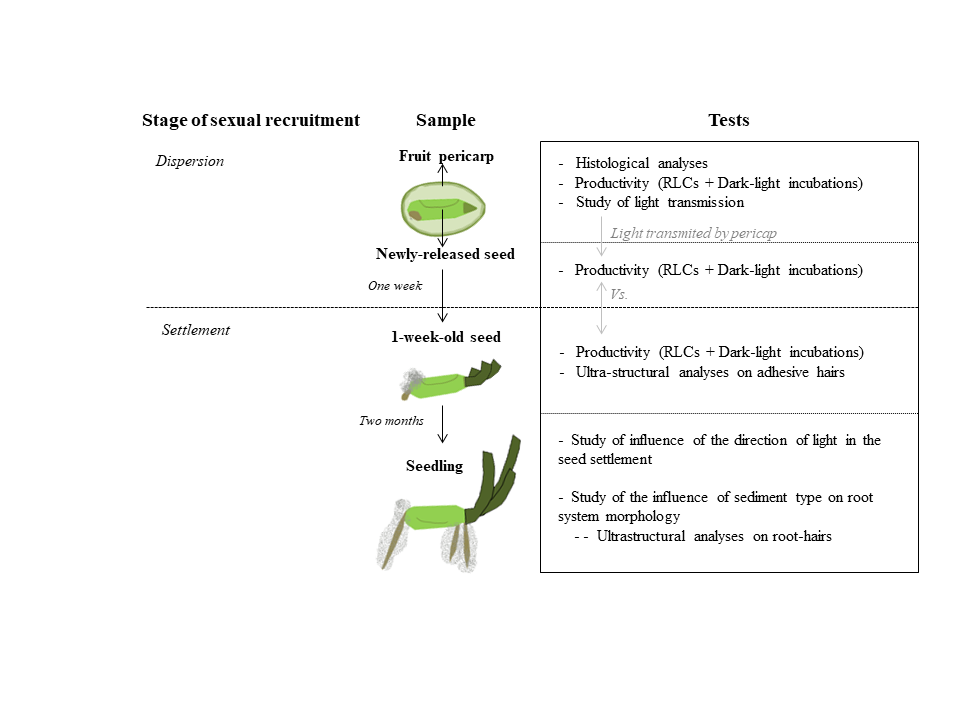

Supplement: S1 Fig — The scheme indicates the recruitment stages of the Posidonia oceanica seeds (seed inside fruit pericarp, newly-released and 1-week-old seeds) and the tests used in each experiment. (TIF) [file pone.0207345.s001.tif]

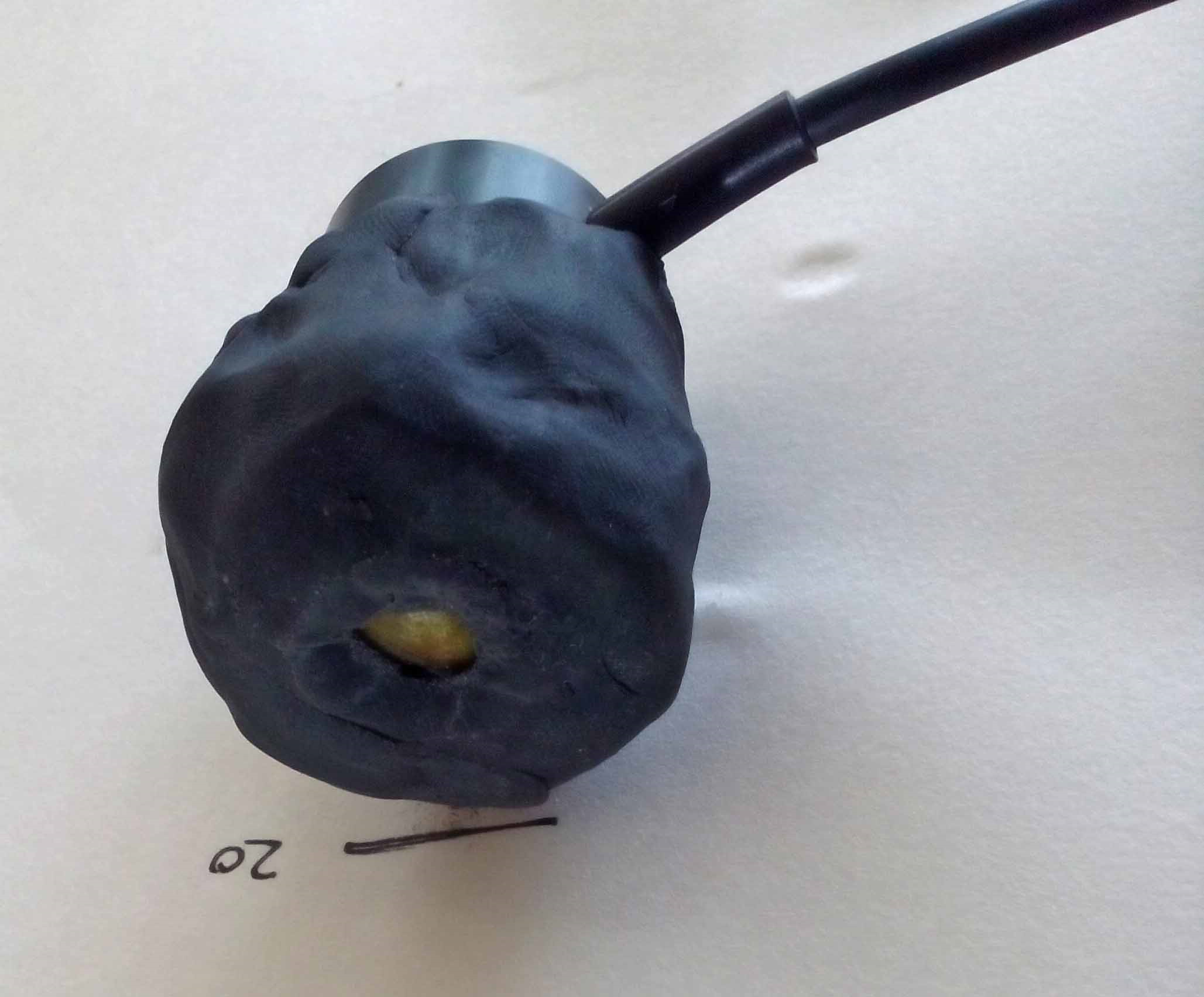

Supplement: S2 Fig — In this image, the probe, with the fruit pericarp coupled inside it, was situated 20 cm from the light source. (TIF) [file pone.0207345.s002.tif]

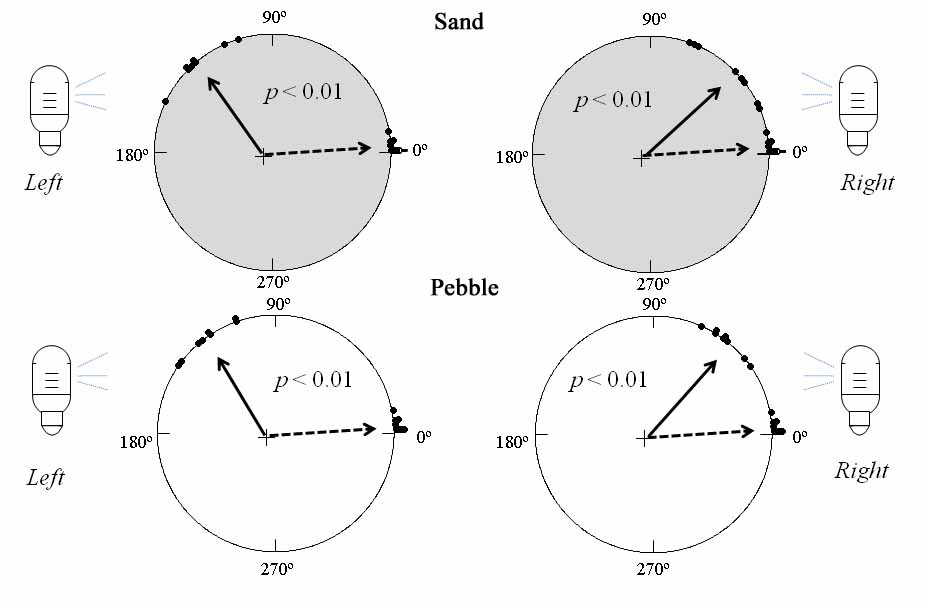

Supplement: S3 Fig — Dashed lines indicate the angle of the orientation of the seeds obtained when light came from the top. Solid lines indicate the average angle of orientation of the seeds obtained in each treatment in the seedlings cultured with lateral lights (right: 180°; left: 0°). Black points indicate the angle of orientation obtained in each sample. Significant differences between the top and lateral lights (right and left) were indicated as p <0.01 in each treatment. (TIF) [file pone.0207345.s003.tif]

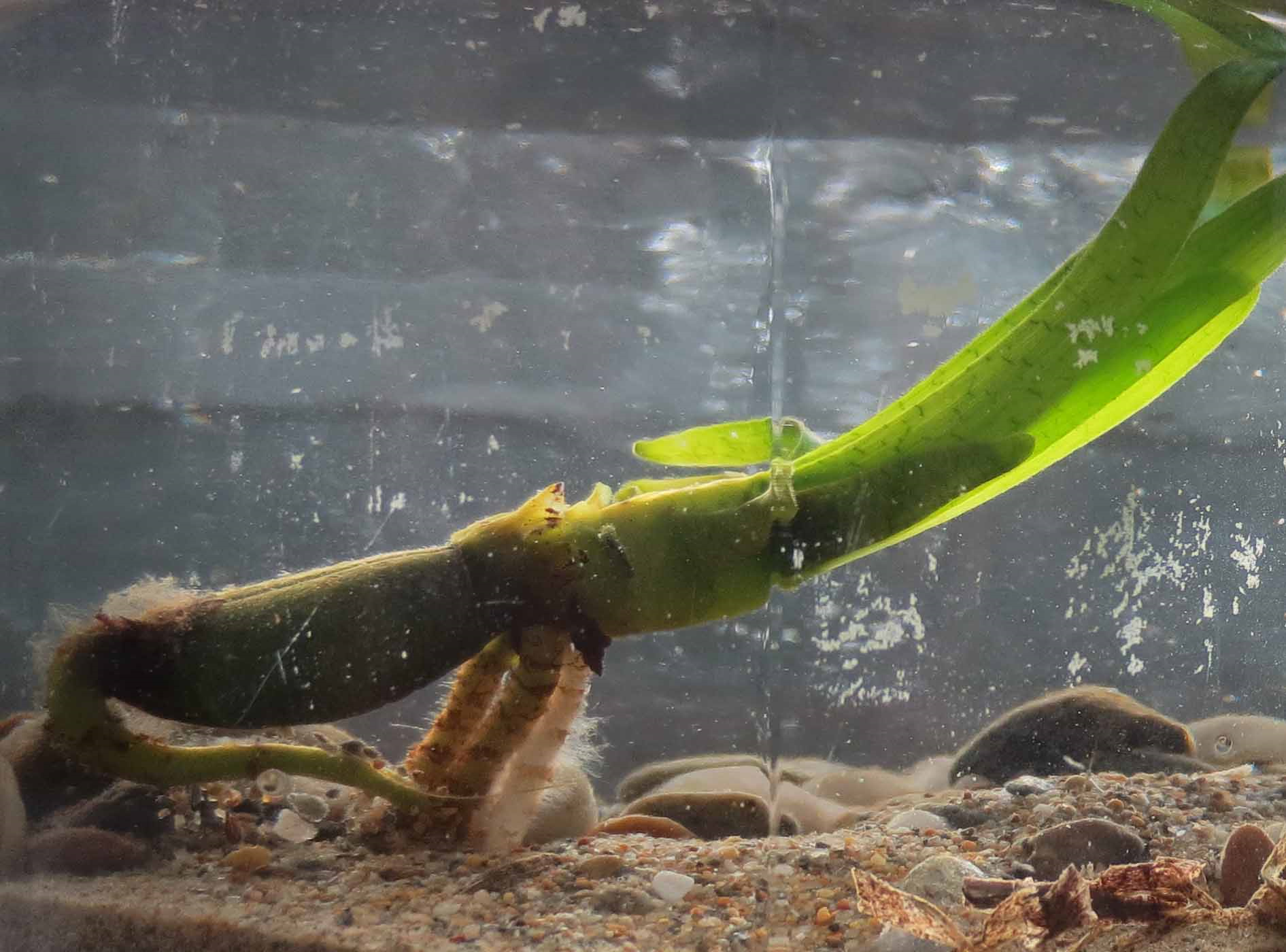

Supplement: S4 Fig — (TIF) [file pone.0207345.s004.tif]
