# Supplementary material for: Understanding the sexual recruitment of one of the oldest and largest organisms on Earth, the seagrass Posidonia oceanica
Source: PLoS One. 2018 Nov 16;13(11):e0207345. doi: 10.1371/journal.pone.0207345 (PMC6239318; doi:10.1371/journal.pone.0207345)
Supplement: S1 Table — (DOCX) [file pone.0207345.s005.docx]

**S1 Table.** Ranges of % used in the experiment of sediment type influence on the root system morphology to determine anchorage success and the density of adhesive hairs obtained after 1 month of seedling development.

| **Anchorage success of the root system** | | **Density of adhesive hairs** |
| --- | --- | --- |
| 0 | The seedling was floating in seawater | The presence of hairs was null |
| 25% | The seedling was anchored only to the sediment by one root | Hairs were hardly observable and whitish |
| 50% | The seedling was anchored to the sediment by the primary root and at least by a secondary root | Hairs were clearly distinguishable and whitish |
| 75% | The seedling was anchored by the primary root and more than half of the secondary root. | Hairs were very clearly distinguishable and yellowish |
| 100% | The seedling used the primary root and all the secondary roots to anchor to sediment | Hairs were very dense and brownish |
